# Synthesis of Aliphatic Polyanhydrides with Controllable and Reproducible Molecular Weight

**DOI:** 10.3390/pharmaceutics14071403

**Published:** 2022-07-04

**Authors:** Radhakanta Ghosh, Yuvaraj Arun, Peter Siman, Abraham J. Domb

**Affiliations:** 1 The Alex Grass Center for Drug Design and Synthesis and Center for Cannabis Research, School of Pharmacy, Institute of Drug Research, The Hebrew University of Jerusalem, Jerusalem 9112001, Israel; onlyradhakanta@gmail.com (R.G.); arun.y.chem@gmail.com (Y.A.); 2 Intragel, Wadi El Haj, Nazareth 17111, Israel; peter@intra-gel.com

**Keywords:** polyanhydrides, controlled molecular weight, melt polycondensation, drug delivery

## Abstract

Polyanhydrides have been synthesized for decades by melt-polycondensation of diacid monomers and 5 to >10 times mole excess acetic anhydride to diacid monomers to form polymers with a polydispersity ranging from 2.5 to 6 and low reproducibility. Hydrophobic segments in polyanhydrides are beneficial to hinder the characteristic hydrolytic cleavage of an anhydride bond that provides stable polyanhydrides at room temperature. The objective of this work is to synthesize aliphatic polyanhydrides with various hydrophobic segments, controllable and reproducible molecular weight, and low polydispersity that are essential for potential use as drug carriers. A series of polyanhydrides of suberic, azelaic, sebacic, and dodecanedioic acids with controlled molecular weight, reduced polydispersity, and standard deviation of molecular weights, have been synthesized. All synthesized polyanhydrides were thoroughly characterized by NMR, Fourier transform infrared spectroscopy, and gel permeation chromatography. Molecular weights of the synthesized polyanhydrides are highly controllable, depending on the degree of activation of the dicarboxylic acid monomers, i.e., the amount of acetic anhydride used during synthesis. Polyanhydrides have been synthesized in triplicate by melt-polycondensation, using various mole ratios of acetic anhydride to diacids. The standard deviation of the molecular weights of the polyanhydrides is minute when using 1 equivalent of acetic anhydride during the activation of dicarboxylic acids, whereas if excess acetic anhydride is used, the standard deviation is very high. The effect of safe and natural inorganic catalysts, Calcium oxide, Zinc oxide, and Calcium carbonate on polymerization is also studied. As-synthesized poly(sebacic acid) can offer convenience to use in controlled drug delivery applications. In vitro drug release study using Temozolamide (TMZ), a medication used to treat brain tumors such as glioblastoma and anaplastic astrocytoma, shows 14% TMZ release after the first hour and 70% release over one day from the poly(sebacic acid) wafers.

## 1. Introduction

Polyanhydrides have been widely explored as biodegradable polymers for the last four decades [1,2,3,4,5,6,7,8,9,10,11,12]. In 1909, Bucher and Slade first synthesized polyanhydrides upon heating isophthalic acid and terephthalic acid in presence of acetic anhydride [1]. In 1983, Langer used polyanhydrides for the first time as biodegradable carriers in various medical devices by identifying and accomplishing the hydrolytic instability of polyanhydrides for controlled drug delivery applications [1]. Polyanhydrides degrade under a wide range of physiological conditions that enable controlled release throughout the degradation process [3,4,5].

Polyanhydrides in the form of slabs, films, rods, microspheres and nanospheres, have been used as therapeutic delivery devices [1,2,3,4,5,6,7,8,9,10,11,12]. Gliadel wafers are in clinical use for the delivery of carmustine (bis-chloroethylnitrosourea, BCNU) for the treatment of brain cancer. These wafers are placed in the tumor site in the brain, after surgical removal of the solid tumor. This device allowed the localized and controlled delivery of BCNU over several weeks that provides high drug concentration in the tumor site with little systemic distribution which reduces BCNU side effects [6,7]. Local delivery antibiotic implant was constructed with fatty acid based polyanhydride for the treatment of osteomyelitis [10,11]. The implant was used to deliver gentamicin sulfate. Polyanhydrides used in other drug delivery applications include local anesthetics, anticancer agents, anticoagulants, delivery of DNA in gene therapy and other large molecules such as proteins, and delivery of neuroactive drugs. The drug delivery of polyanhydrides has been reviewed [4,5].

Polyanhydrides are straightforward and inexpensive to synthesize or scale up and can be manipulated easily to meet desirable properties. They have a hydrophobic backbone with hydrolytically labile anhydride linkages. Therefore, polyanhydrides exhibit a short shelf-life, and the anhydride linkage can easily hydrolyze into dicarboxylic acids monomers in the presence of moisture [13,14,15,16,17]. The quick degradation of polyanhydrides sometimes presents difficulties during handling. Accordingly, these polymers must be stored and handled under special conditions such as a moisture-free atmosphere and low temperatures [18,19]. Due to their quick degradation and limited mechanical properties, their primary use has been restricted to short-term controlled delivery of bioactive agents [20].

The rate of degradation of polyanhydrides, however, can be controlled depending upon the ratio of their hydrophobic and hydrophilic segments [17,21,22]. The incorporation of ester bonds into anhydride chains is another way to significantly extend the shelf-life and improve the stability of anhydrides. ε-caprolactone based polyanhydride copolymers show elevated hydrolytic stability [23]. Alternating architecture and hydrophobic side chains also hinder hydrolytic cleavage and anhydride interchange in poly(ester-anhydrides), which provides stable polyanhydrides at room temperature. Polyanhydrides, synthesized from ricinoleic and sebacic acid with alternating ester-anhydride bonds, are stable at 25 °C for more than 18 months [19]. In this study, the authors have shown that alternating architecture has hydrophobic side chains that help to improve stability by hindering hydrolytic cleavage and anhydride interchange.

The molecular weight and molecular weight distribution of polyanhydride strongly affect the polymer properties, including: mechanical properties, solubility and viscosity of a polymer solution, precipitation efficiency from an antisolvent, viscosity of pasty injectable polymer, melt molding and degradation rate and drug release, Thus, it is essential to obtain polyanhydrides with reproducible and controlled molecular weight for medical as well as other uses.

Polyanhydrides are synthesized using simplified methods such as melt polycondensation, solution polymerization, use of coupling agents, and ring-opening polymerization [2]. Among them, polycondensation of dicarboxylic acids in the presence of a large excess of refluxing acetic anhydride is the most widely used protocol for the synthesis of polyanhydrides. For example, Niewolik et al. synthesized a polyanhydride using 1:10 *w*/*v* acetic anhydride [24]; Kelly et al. synthesized several polyanhydrides using 1:45 *w*/*v* acetic anhydride [25]; Chu et al. polymerized sebacic acid and other diacids using 1:10 *w*/*v* acetic anhydride [26]; Herrera et al. synthesized poly(azelaic anhydride) by microwave irradiation (5 min) using a 1:3 *w*/*v* relation of solid dicarboxylic acid to acetic anhydride [27]. Early reports on polyanhydride synthesis used 1:5 *w*/*v* diacid to an anhydride ratio [19,22]. Polymers with different uncontrollable molecular weight were obtained using all these methods, depending on reaction conditions.

Polyanhydrides facilitate new potential uses as injectable systems in the medical field, due to their viscous and low melting nature. The injectable formulation can be simply squeezed out using a needle that creates a deposit under the aqueous atmosphere and can steadily release a loaded drug. This permits localized drug delivery with minimal invasion. Carmustine, an anticancer agent to cerebral tumor sites, is delivered using a Gliadel wafer that is an approved polyanhydride copolymer of carboxyphenoxy propane and sebacic acid [7]. Polyanhydride based particles have also been extensively used in many formulations for effective drug delivery [9,12,28].

The synthetic method certifying proper control over polyanhydride architecture is key for superior biodegradable polymers. Hence, the objective of this work is to set up a method for the synthesis of polyanhydrides with a desired and controlled molecular weight by reacting diacids with a mole equivalent amount or less acetic anhydride per carboxylic acid residue, and to form polyanhydrides with a chain length correlated to the mole ratio of carboxylic acid groups to acetic anhydride molecules. In all previous publications a large excess of acetic anhydride of 3 to 20 *w*/*v* times was used, but this formed polymers of uncontrolled and diverse molecular weights. In this work, dicarboxylic acids (suberic, azelaic, sebacic, and dodecanedioic acids) were reacted with one equivalent of acetic anhydride per carboxylic acid. They formed a polymer with a reproducible molecular weight within the range of the molecular weight obtained when using 1:5 or 1:10 *w*/*v* excess of acetic anhydride under the same conditions. The molecular weight is proportionally reduced, as the mole ratio of dicarboxylic acid to acetic anhydride increases.

## 2. Experimental Section

### 2.1. Materials

Diacids such as Suberic acid (98%, Sigma-Aldrich, Rehovot, Israel), Azelaic acid (98%, Sigma-Aldrich, Rehovot, Israel), Sebacic acid (99% pure; Sigma-Aldrich, Rehovot, Israel), and Dodecanedioic acid (99%, Sigma-Aldrich, Rehovot, Israel), were used as received. Acetic anhydride was purchased from Merck, Israel and used as received. All solvents and reagents (analytical-grade) were purchased (Sigma–Aldrich, Rehovot, Israel or Bio-Lab, Jerusalem, Israel) and used without further purification.

### 2.2. Spectral Analysis

^1^H spectra were obtained on a Varian 300 MHz NMR spectrometer in tubes with 5 mm external diameter. CDCl_3_ containing tetramethylsilane was used as a solvent and shift reference. Fourier transform infrared (FTIR) spectroscopy was performed using a Smart iTR ATR sampling accessory for Nicolet iS10 spectrometer with a diamond crystal (Thermo Scientific, Waltham, MA, USA).

### 2.3. Molecular Weight Determination

The molecular weights of the synthesized polyanhydrides were determined by a gel permeation chromatography (GPC) system consisting of a Waters 1515 isocratic HPLC pump with a Waters 2410 refractive index detector, a Waters 717 plus autosampler, and a Rheodyne (Cotati, CA, USA) injection valve with a 20 μL-loop. The samples were eluted with CHCl_3_ (HPLC grade) through linear Styragel HR5 column (Waters) at a flow rate of 1 mL/min. The molecular weights were determined relative to polystyrene standards (Polyscience, Warrington, PA, USA).

### 2.4. Synthesis of Polyanhydrides Using Acetic Anhydride

All polyanhydrides were synthesized by reflux of respective diacids with different amounts of acetic anhydride followed by the polymerization through melt condensation.

#### 2.4.1. Synthesis of Poly(sebacic Acid)

The sebacic acid (2.0 g) was melted at 140 °C under nitrogen atmosphere. Then different amounts (5 equiv., 1.0 equiv., 0.75 equiv., 0.50 equiv., 0.25 equiv., and 0.10 equiv.) of acetic anhydride with respect to carboxylic acid groups were added to the molten dicarboxylic monomer and refluxed at 140 °C for 1 h. Excess acetic anhydride or acetic acid was evaporated. The residue was then subjected to melt condensation at 160 °C under vacuum (~10 m bar) for 4 h. In addition, a reaction with 0.1 equiv. acetic anhydride was kept overnight for melt condensation. The polymers obtained were characterized by NMR, FTIR, and GPC. ^1^H NMR (300 MHz, Chloroform-d) δ 2.45 (t, J = 7.4 Hz, 4H), 1.65 (p, J = 7.1 Hz, 4H), 1.44–1.22 (m, 8H); FTIR (cm^−1^) 2927, 2913, 2850, 1808, 1741, 1471, 1411, 1358, 1035.

#### 2.4.2. Extension to Other Dicarboxylic Acids

In a typical synthesis, 2 g of each dicarboxylic monomer (suberic acid, azelaic acid, and dodecanedioic acids) was melted at 140 °C under nitrogen atmosphere. Then, different amounts (5 equiv., 1.0 equiv., 0.75 equiv., 0.5 equiv., and 0.25 equiv.) of acetic anhydride with respect to carboxylic acid groups were added to every molten dicarboxylic monomer and refluxed at 140 °C for 1 h. Excess acetic anhydride or acetic acid was evaporated. The residue was then subjected to melt condensation at 160 °C under vacuum (~10 m bar) for 4 h and characterized by NMR, FTIR, and GPC.

### 2.5. Synthesis of Poly(sebacic Acid) Using Other Catalysts

Poly(sebacic acid) was also synthesized by heating sebacic acid with other catalysts followed by polymerization through melt condensation [29]. The sebacic acid (25 g, 123.6 mmol, 1 equiv.) was activated by heating at 140 °C with acetic anhydride (7.0 mL, 74.1 mmol, 0.3 equiv.). Five different types of polymerizations were achieved using activated sebacic acid (2.0 g, 9.9 mmol) with different catalysts such as toluene (2.0 mL, 1:1 *w*/*v*), CaO (5.6 mg, 0.099 mmol, 1 mol%), ZnO (8.1 mg, 0.099 mmol, 1 mol%) or CaCO_3_ (9.9 mg, 0.099 mmol, 1 mol%), and neat condition (without any catalyst) as a control experiment. Each reaction mixture was polymerized by melt condensation at 160 °C for 4 h under vacuum (10 mbar) with constant stirring. The polymerization was monitored by NMR, GPC, and FT-IR.

### 2.6. Wafer Preparation of 50% Tmz, 50% Poly(Sebacic Acid), 2 Coating Layers

35 mg of poly(sebacic acid) was dissolved into 0.25 mL of dichloromethane (DCM). 70 mg of TMZ was inserted into the polymer solution and stirred until complete evaporation of the DCM and formation of a powder. The second coating was applied by adding the coated powder in a new poly(sebacic acid) solution (35 mg of polymer in 0.25 mL DCM) and mixing for evaporation of most of the DCM. Heptane (5 mL, 1:20) was added and was mixed well. Then particles were allowed to precipitate, the solvent was decanted, and fresh heptane was added (5 mL). Heptane was decanted again, and the particles were dried in room air. Wafers of 10 mg each, 3 mm in diameter, were prepared by compression molding at 1.5 tons.

### 2.7. In Vitro Drug Release

Poly(sebacic acid) was investigated for its in vitro drug release properties using TMZ as a model drug. TMZ wafers (10 mg) were added into 5 mL of acetate buffer pH 3.5 as a release medium, incubated, the stirred at 37 °C. The solutions were taken out after 1 h, 24 h, 4 days, and 7 days without disturbing the formulation. After removing the release medium, a fresh buffer solution was added at all time points. TMZ content in the released samples was determined by HPLC. All the experiments were done in triplicate.

### 2.8. HPLC Analysis for TMZ Release Study

HPLC analysis was performed on a LiChrospher^®^ RP-C18 (5 µm) column packed in LichroCART 250- 4 HPLC cartridges (Merck, Germany). The chromatographic system used was a Merck Hitachi Lachrom HPLC system equipped with a UV detector (Model Lachrom L7400). The mobile phase consisted of an aqueous phase (0.5% of glacial acetic acid in double distilled water) and organic phase (methanol) in a ratio of 9:1 used in isocratic mode. Sample injection volume was 50 µL. The analysis was carried out at an auto-sampler temperature of 17 °C, column at room temperature, and flow rate of 1.0 mL/min. The effluent was monitored on the UV detector attached to the HPLC system at a wavelength of 254 nm. TMZ showed a retention time of 6.32 min. under these conditions. Calibration plots for TMZ were prepared in a concentration range of 0.2–100 µg/mL.

## 3. Results and Discussion

### 3.1. Synthesis of Polyanhydrides

**Scheme 1:**
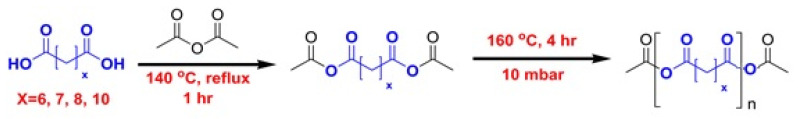
Synthesis of polyanhydrides from different aliphatic dicarboxylic acid.

A series of polyanhydrides was synthesized through a solvent-free melt polycondensation process using different dicarboxylic acids. The detailed synthetic methodology is given in Scheme 1. At first each diacid was activated using acetic anhydride, and then the polyanhydrides were obtained by melt condensation. If an excess amount of acetic anhydride was taken compared to diacids, it had to be evaporated to dryness under vacuum at 70 °C. The clear residue was further polymerized by melt condensation at 160 °C for 4 h under vacuum (10 mbar) with constant stirring, yielding polyanhydrides as the final polymer.

### 3.2. FTIR Study

**Figure 1:**
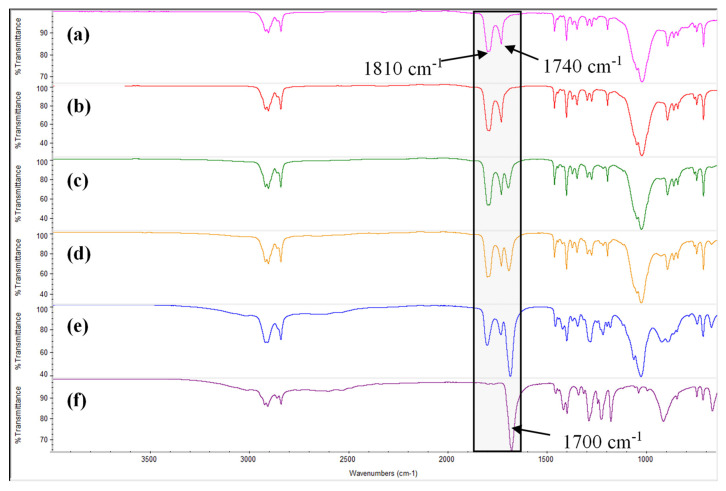
FT−IR spectra of poly(sebacic acid) synthesized using (**a**) 5 equiv. (**b**) 1 equiv. (**c**) 0.75 equiv. (**d**) 0.5 equiv. and (**e**) 0.25 equiv. of acetic anhydride; (**f**) sebacic acid. Polyanhydrides synthesized in triplicate at 160 °C under vacuum (~10 m bar) for 4 h.

The FTIR spectra of poly(sebacic acid) and pure sebacic acid are presented in Figure 1. The characteristic stretching frequency of the C=O (acid) of sebacic acid arises at 1700 cm^−1^. The diacid was polymerized, and polyanhydrides were confirmed by the characteristic bands at 1810 cm^−1^ and for 1740 cm^−1^ for C=O (anhydride) of poly(sebacic acid). FTIR spectra of the synthesized polyanhydrides show that the C=O stretching frequency of the acid group at ~1700 cm^−1^ decreases with the increase in the acetic anhydride to acid ratio, while the anhydride bond stretching peak at ~1815 cm^−1^ rises, as shown for the polymerization of sebacic acid (Figure 1) [30].

The ratios of peak height of anhydride with respect to acid show that it gradually increases from 0.25 equivalents to 1 or 5 equivalents of used acetic anhydride. When 0.25 equiv. of acetic anhydride is used, the weak stretching frequencies at ~1815 cm^−1^ and ~1740 cm^−1^, and strong frequency around ~1700 cm^−1^ correspond to anhydride and carboxylic acid bonds [30]. This indicates the presence of acids and partial conversion to anhydrides due to the formation of polyanhydrides with low molecular weight. When increasing the quantity of acetic anhydride from 0.25 equiv. to 1.0 equiv., an increase in the anhydride bond intensity and a decrease in the acid bond intensity in FTIR spectra were observed. When 1 equiv. of acetic anhydride is used, almost all the acids are converted into anhydrides.

### 3.3. NMR Study

**Figure 2:**
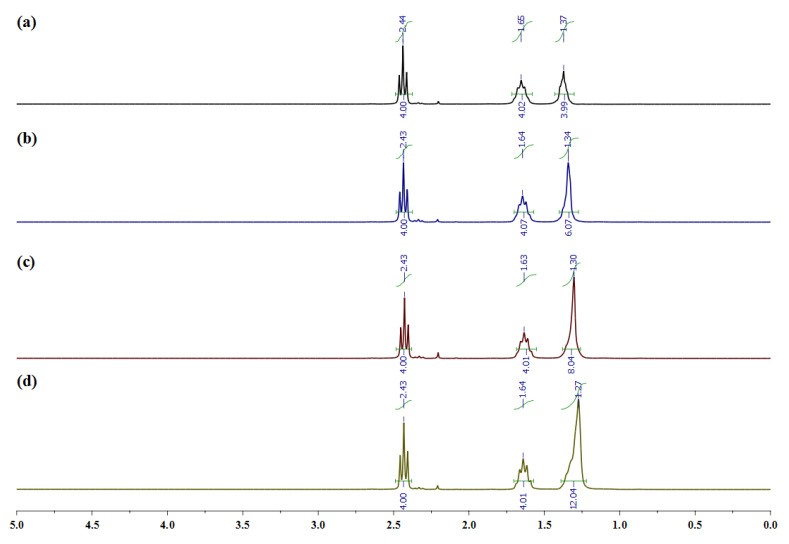
^1^H NMR spectra of (**a**) poly(suberic acid), (**b**) poly(azelaic acid), (**c**) poly(sebacic acid) and (**d**) poly(dodecanedioic acid). Polyanhydrides synthesized in triplicate using 1 equiv. of acetic anhydride with respect to the acid groups, at 160 °C under vacuum (~10 m bar) for 4 h.

The synthesized polyanhydrides were characterized by NMR to confirm the structure (Figure 2). ^1^H NMR spectra of poly(suberic acid) (1.37 (s, 4 H), 1.65 (4 H), 2.44 (t, 4 H)), poly(azelaic acid) (1.34 (s, 6 H), 1.64 (4 H), 2.43 (t, 4 H)), poly(sebacic acid) (1.30 (s, 8 H), 1.63 (4 H), 2.43 (t, 4 H)), and poly(dodecanedioic acid) (1.27 (s, 12 H), 1.64 (4 H), 2.43 (t, 4 H)) were synthesized using 1 equiv. of acetic anhydride with respect to the acid groups [31]. These NMR studies confirm the successful formation of each polyanhydride.

### 3.4. Molecular Weight Measurement by GPC

**Figure 3:**
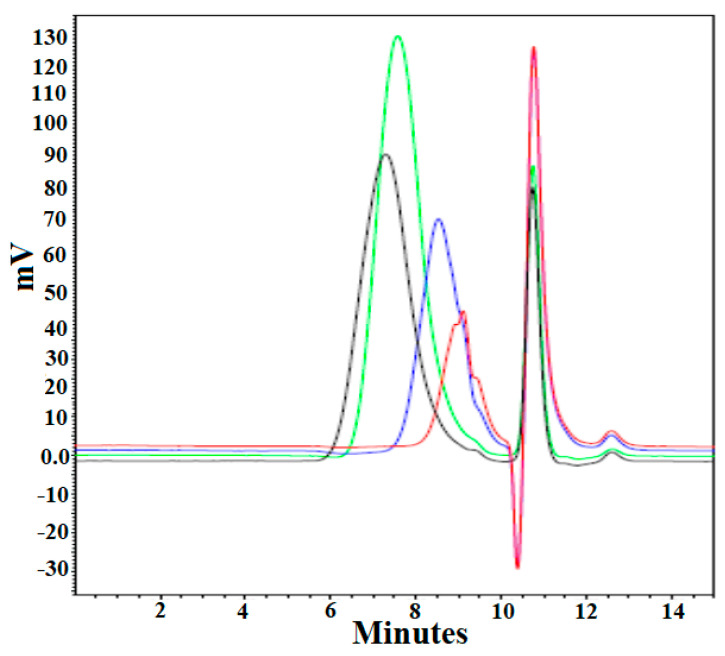
Overlay of GPC chromatograms of PSA synthesized by melt condensation of sebacic acid in the presence of 0.25 (red), 0.5 (blue), 1.0 (green) and 5.0 (black) (X axis is elusion time in minutes, Y axis is the intensity of absorption which translates to content).

**Figure 4:**
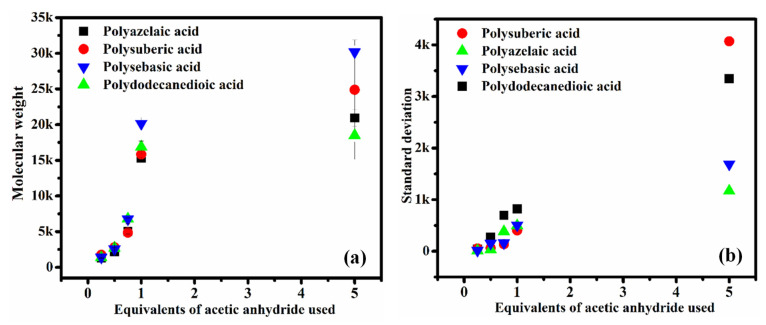
(**a**) Variation of polyanhydride weight average molecular weights and (**b**) variation of standard deviation as a function of acetic anhydride to diacid monomer molar ratio. Polyanhydrides synthesized in triplicate at 160 °C under vacuum (~10 m bar) for 4 h.

The molecular weight of the synthesized polyanhydrides in triplicate was analyzed by GPC. Figure 3 shows the GPC overlay chromatograms of the polymers resulted from the synthesis of PSA prepared by melt condensation using mole ratios of 0.25, 0.5, 1.0 and 5.0 acetic anhydride to carboxylic acid moieties of the diacid monomers. The molecular weight of each polyanhydride gradually increases when adding more acetic anhydride from 0.25 equiv. to 1 or 5 equivalents (Figure 4a). The dicarboxylic acid monomers are converted into polymers with weight average molecular weights (M_w_) of ~1500 Da, ~2600 Da, ~5500 Da, ~16,000 Da by using 0.25 equiv., 0.5 equiv., 0.75 equiv., and 1 equiv. of acetic anhydride, respectively.

The study reveals that 1.0 equiv. of acetic anhydride is sufficient to obtain almost similar molecular weight, when 5 equiv. (excess) acetic anhydride is employed. However, 0.1 equiv. acetic anhydride is insufficient for the synthesis of polyanhydrides even after overnight polymerization. Control over the molecular weight of polyanhydride depending upon the acetic anhydride used is given in Figure 4b, in which the variation of standard deviation of the M_w_ of the synthesized polyanhydrides in triplicate were plotted against the used acetic anhydride during the activation of dicarboxylic acid monomers. The polymers of dicarboxylic acid have a control molecular weight with standard deviation of <500 by using 1 equiv. or less of acetic anhydride, whereas if excess acetic anhydride is used, the standard deviation is very high (~2500). The polydispersity of the polyanhydrides synthesized with 1 equivalent of acetic anhydride was in the range of 1.3 to 2.0, while the polymers synthesized using 5 equivalents of acetic anhydrides possess polydispersity between 2.5 and 5.5.

The controlled molecular weight and narrow polydispersity are related to the fact that when reacting the diacid monomers with a mole equivalent or less of acetic anhydride, all acetic anhydride is used for the formation of the activated diacid anhydride with some carboxylic acid groups remaining without acetylation. The number of free carboxylic acid groups remaining in the polymerization system is proportionate to the mole ratio of the starting ratio of acetic anhydride to carboxylic acids; the less acetic anhydride used, more free carboxylic acids are in the polymerization system. These free carboxylic acids serve as chain terminations, resulting in a controlled and lower molecular weight that is proportional to the acetic anhydride use. When excess acetic anhydride is used, the reaction solution is much less viscous which allows oligomer formation with no carboxylic acid terminators. Thus, the molecular weight is dependent on the polymerization conditions.

### 3.5. Effect of Inorganic Catalysts on Molecular Weight

**Figure 5:**
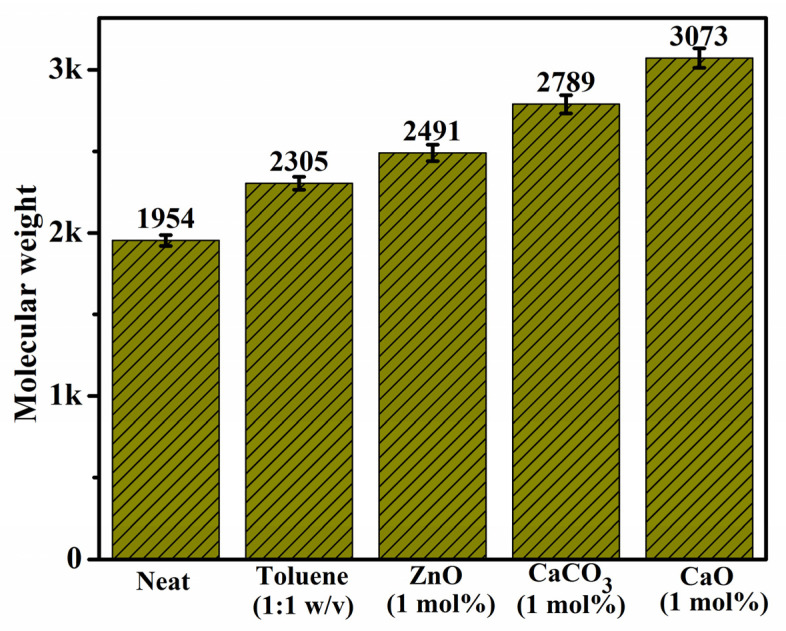
Weight average molecular weights of poly(sebacic acid) using different catalysts, analyzed by GPC. Sebacic acid was polymerized by heating with 0.3 equivalents of acetic anhydride along with 1% *w*/*w* catalyst powder and no solvents. Polyanhydrides synthesized in triplicate at 160 °C under vacuum (~10 m bar) for 4 h.

Poly(sebacic acid) was also synthesized in the presence of polymerization catalysts [29]. Sebacic acid was activated by heating with acetic anhydride (0.3 equiv.). The following agents were used: toluene (1:1 *w*/*v*), CaO (1 mol%), ZnO (1 mol%), and CaCO_3_ (1 mol%). Neat condition (without any catalyst) was used as a control experiment. The molecular weight was determined by GPC (Figure 5).

An increase in M_w_ was obtained compared to the polymerization conducted without catalysts. CaO was superior among the used catalysts. FT-IR spectra of the synthesized poly(sebacic acid) confirmed incomplete polymerization of the diacids as a peak at 1700 cm^−1^ is visible, although the size ratio decreases with the increase in molecular weight.

### 3.6. In Vitro Drug Release Studies

**Figure 6:**
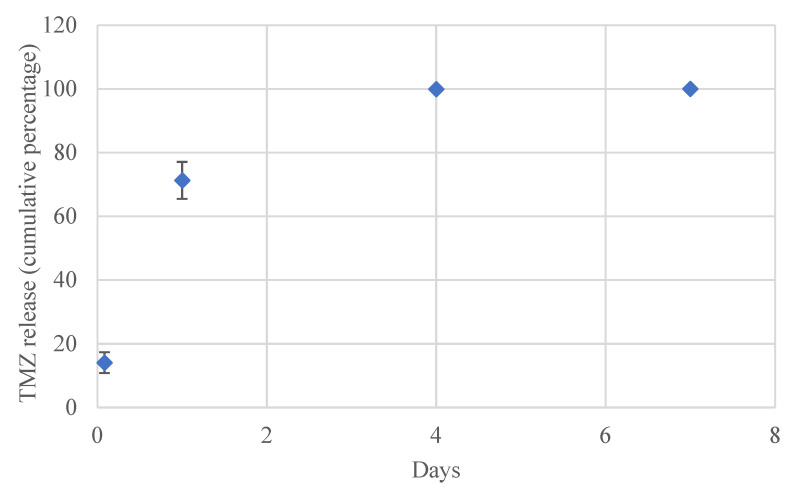
In vitro release of Temozolamide in acetate buffer of pH 3.5 at 37 °C. The amount of Temozolamide was calculated using HPCL. The percentage errors are calculated from an average of three observations.

The synthesized poly(sebacic acid) was examined for its in vitro drug release pattern using temozolamide (TMZ), a highly water soluble anticancer drug for treating brain tumors. Wafers containing 50% *w*/*w* TMZ were prepared by first solvent coating of the TMZ particles with PSA, followed by compression molding into a tablet. Tablets made from direct mixing of PSA and TMZ powders resulted in an immediate release of TMZ. The in vitro release was conducted at pH 3.5, due to the instability of TMZ at pH > 4. The results reveal that the TMZ was released from the PSA wafers during 4 days. 14% was released at the first hour, and 70% of the drug was released over one day. The in vitro release of TMZ in acetate buffer of pH 3.5 at 37 °C result is given in Figure 6. This pH was selected due to the limited stability of TMZ at pH > 3.5. The weight loss of the polymer carrier under the same release study, after 1, 4, and 7 days was, 5, 20 and 35% *w*/*w*.

## 4. Conclusions

**Figure A1:**
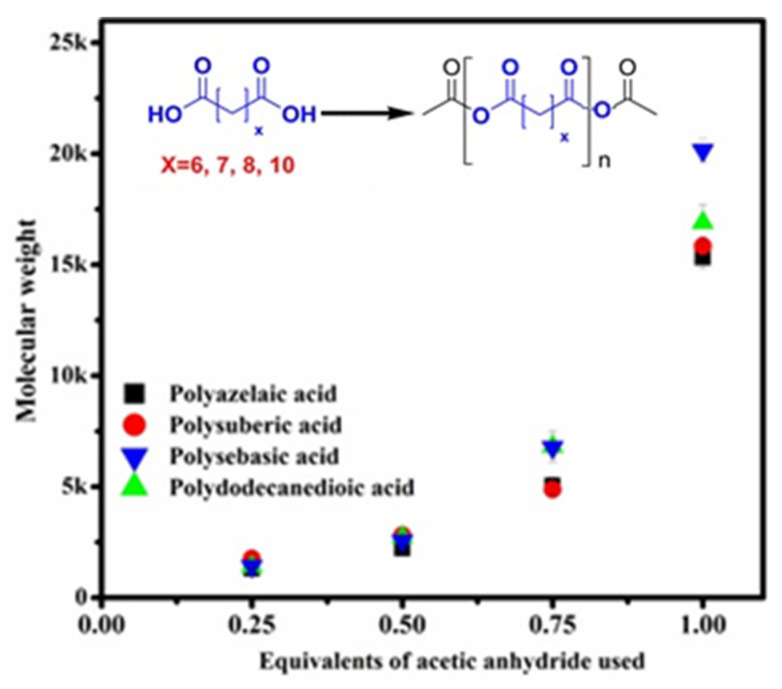
Table of Contents Figure.

An effective route for the synthesis of aliphatic polyanhydride, made from a series of dicarboxylic acids with controlled molecular weight and narrow polydispersity, is described (Appendix A, Figure A1). One equivalent of acetic anhydride to acid groups in the diacid monomers is sufficient to obtain a polymer with controlled molecular weight and narrow polydispersity. This is probably due to carboxylic acid end groups that terminate the polymerization. Thus, as the molar ratio of acetic anhydride to carboxylic acid is below 1, more carboxylic acid groups exist in the polymerization system that serve as terminators, resulting in a reduction in the molecular weight of the polymers. When toluene, CaO, ZnO, and CaCO_3_ are added to the polymerization, a higher molecular weight is obtained compared to polymerization conducted without a catalyst. The molecular weights of the synthesized polymers in this protocol are highly controllable, depending upon the degree of activation of the monomers. The synthesized injectable pasty poly(sebacic acid) was analyzed for in vitro drug release using Temozolamide. It shows a 14% drug release at the first hour and 70% release over one day from the poly(sebacic acid) wafers. Hence, this route presents the possibility to produce aliphatic polyanhydride with controlled molecular weight for possible use in the preparation of degradable disposable medical supplies.

## Appendix A. Table of Contents

Biodegradable aliphatic polyanhydrides from diverse dicarboxilic acids with controlled molecular weight and less standard deviation are synthesized using the various quantities of acetic anhydride by melt polycondensation. This route brought forward an idea for producing aliphatic polyanhydride with targeted molecular weight for better use in the preparation of degradable disposable medical supplies.

## References

[1] Rosen H.B., Chang J., Wnek G.E., Linhardt R.J., Langer R. (1983). Bioerodible polyanhydrides for controlled drug delivery. Biomaterials.

[2] Kumar N., Langer R.S., Domb A.J. (2002). Polyanhydrides: An overview. Adv. Drug Deliv. Rev..

[3] Göpferich A., Tessmar J. (2002). Polyanhydride degradation and erosion. Adv. Drug Deliv. Rev..

[4] Basu A., Domb A.J. (2018). Recent Advances in Polyanhydride Based Biomaterials. Adv. Mater..

[5] Arun Y., Ghosh R., Domb A.J. (2021). Biodegradable Hydrophobic Injectable Polymers for Drug Delivery and Regenerative Medicine. Adv. Funct. Mater..

[6] Champeaux C., Weller J. (2020). Implantation of carmustine wafers (Gliadel^®^) for high-grade glioma treatment. A 9-year nationwide retrospective study. J. Neurooncol..

[7] Brem H., Gabikian P. (2001). Biodegradable polymer implants to treat brain tumors. J. Control. Release.

[8] Darling R., Senapati S., Christiansen J., Liu L., Ramer-Tait A.E., Narasimhan B., Wannemuehler M. (2020). Polyanhydride Nanoparticles Induce Low Inflammatory Dendritic Cell Activation Resulting in CD8^+^ T Cell Memory and Delayed Tumor Progression. Int. J. Nanomed..

[9] Zada M.H., Kubek M., Khan W., Kumar A., Domb A.J. (2019). Dispersible hydrolytically sensitive nanoparticles for nasal delivery of thyrotropin releasing hormone (TRH). J. Control. Release.

[10] Krasko M.Y., Golenser J., Nyska A., Nyska M., Brin Y.S., Domb A.J. (2007). Gentamicin extended release from an injectable polymeric implant. J. Control. Release.

[11] Ramot Y., Steiner M., Amouyal N., Lavie Y., Klaiman G., Domb A.J., Nyska A., Hagigit T. (2021). Treatment of contaminated radial fracture in Sprague-Dawley rats by application of a degradable polymer releasing gentamicin. J. Toxicol. Pathol..

[12] Brenza T.M., Schlichtmann B.W., Bhargavan B., Vela Ramirez J.E., Nelson R.D., Panthani M.G., McMillan J.M., Kalyanaraman B., Gendelman H.E., Anantharam V. (2018). Biodegradable polyanhydride-based nanomedicines for blood to brain drug delivery. J. Biomed. Mater. Res. A.

[13] Jaszcz K. (2014). Effect of Basic Factors of Preparation on Characteristics, Hydrolytic Degradation, and Drug Release from Poly(ester-anhydride) Microspheres. Int. J. Polym. Mater..

[14] Korhonen H., Hakala R.A., Helminen A.O., Seppala J.V. (2006). Synthesis and Hydrolysis Behaviour of Poly(ester anhydrides) from Polylactone Precursors Containing Alkenyl Moieties. Macromol. Biosci..

[15] de Ronde B.M., Carbone A.L., Uhrich K. (2010). Storage stability study of salicylate-based Poly(anhydride-esters). Polym. Degrad. Stab..

[16] Krasko M.Y., Shikanov A., Ezra A., Domb A.J. (2003). Poly(ester anhydride)s prepared by the insertion of ricinoleic acid into poly(sebacic acid). J. Polym. Sci. Part A Polym. Chem..

[17] Krasko M.Y., Domb A.J. (2005). Hydrolytic Degradation of Ricinoleic-Sebacic-Ester-Anhydride Copolymers. Biomacromolecules.

[18] Poetz K.L., Shipp D.A. (2016). Polyanhydrides: Synthesis, Properties, and Applications. Aust. J. Chem..

[19] Zada M.H., Basu A., Hagigit T., Schlinger R., Grishko M., Kraminsky A., Hanuka E., Domb A.J. (2017). Stable polyanhydride synthesized from sebacic acid and ricinoleic acid. J. Control. Release.

[20] Eameema M., Duvvuri L.S., Khan W., Domb A.J. (2014). Polyanhydrides. Natural and Synthetic Biomedical Polymers.

[21] Domb A.J., Maniar M.J. (1993). Absorbable biopolymers derived from dimer fatty acids. J. Polym. Sci. Part A Polym. Chem..

[22] Zada M.H., Basu A., Hagigit T., Schlinger R., Grishko M., Kraminsky A., Hanuka E., Domb A. (2016). Alternating Poly(ester-anhydride) by Insertion Polycondensation. J. Biomacromol..

[23] Hakala R.A., Korhonen H., Meretoja V.V., Seppälä J.V. (2011). Photo-cross-linked biodegradable poly (ester anhydride) networks prepared from alkenylsuccinic anhydride functionalized poly (ε-caprolactone) precursors. Biomacromolecules.

[24] Niewolik D., Krukiewicz K., Cwynar B.B., Ruszkowski P., Jaszcz K. (2019). Novel polymeric derivatives of betulin with anticancer activity. RSC Adv..

[25] Kelly S.M., Mitra A., Mathur S., Narasimhan B. (2020). Synthesis and Characterization of Rapidly Degrading Polyanhydrides as Vaccine Adjuvants. ACS Biomater. Sci. Eng..

[26] Chu I.M., Liu T.H., Chen Y.R. (2019). Preparation and characterization of sustained release system based on polyanhydride microspheres with core/shell-like structures. J. Polym. Res..

[27] Gutiérrez M., Sierra C., Morantes M.A., Herrera A.P. (2016). Synthesis of (azelaic-co-dodecanedioic) polyanhydride by microwave technique. J. Phys. Conf. Ser..

[28] Wafa E.I., Geary S.M., Ross K.A., Goodman J.T., Narasimhan B., Salem A.K. (2019). Single Dose of a Polyanhydride Particle-Based Vaccine Generates Potent Antigen-Specific Antitumor Immune Responses. J. Pharmacol. Exp. Ther..

[29] Domb A.J., Langer R.S. (1987). Polyanhydrides. I. Preparation of high molecular weight polyanhydrides. J. Polym. Sci. Part A Polym. Chem..

[30] Ickowicz D.E., Abtew E., Khan W., Golovanevski L., Steinman N., Weiniger C.F., Domb A.J. (2016). Poly(ester-anhydride) for controlled delivery of hydrophilic drugs. J. Bioact. Compat. Polym..

[31] Albertsson A.C., Lundmark S. (1990). Synthesis, characterization and degradation of aliphatic polyanhydrides. Br. Polym. J..

